# A long lost key opens an ancient lock: *Drosophila* Myb causes a synthetic multivulval phenotype in nematodes

**DOI:** 10.1242/bio.051508

**Published:** 2020-05-04

**Authors:** Paul J. Vorster, Paul Goetsch, Tilini U. Wijeratne, Keelan Z. Guiley, Laura Andrejka, Sarvind Tripathi, Braden J. Larson, Seth M. Rubin, Susan Strome, Joseph S. Lipsick

**Affiliations:** 1 Departments of Pathology, Genetics, and Biology, Stanford University, Stanford, CA 94305-5324, USA; 2 Department of Molecular, Cell, and Developmental Biology, University of California, Santa Cruz, Santa Cruz, CA 95064, USA; 3 Department of Chemistry and Biochemistry, University of California, Santa Cruz, Santa Cruz, CA 95064, USA

**Keywords:** Myb, Development, Evolution, Oncogene, synMuv, Tumor suppressor

## Abstract

The five-protein MuvB core complex is highly conserved in animals. This nuclear complex interacts with RB-family tumor suppressor proteins and E2F-DP transcription factors to form DREAM complexes that repress genes that regulate cell cycle progression and cell fate. The MuvB core complex also interacts with Myb family oncoproteins to form the Myb-MuvB complexes that activate many of the same genes. We show that animal-type *Myb* genes are present in Bilateria, Cnidaria and Placozoa, the latter including the simplest known animal species. However, bilaterian nematode worms lost their animal-type *Myb* genes hundreds of millions of years ago. Nevertheless, amino acids in the LIN9 and LIN52 proteins that directly interact with the MuvB-binding domains of human B-Myb and *Drosophila* Myb are conserved in *Caenorhabditis* *elegans*. Here, we show that, despite greater than 500 million years since their last common ancestor, the *Drosophila melanogaster* Myb protein can bind to the nematode LIN9-LIN52 proteins *in vitro* and can cause a synthetic multivulval (synMuv) phenotype *in vivo*. This phenotype is similar to that caused by loss-of-function mutations in *C. elegans* synMuvB-class genes including those that encode homologs of the MuvB core, RB, E2F and DP. Furthermore, amino acid substitutions in the MuvB-binding domain of *Drosophila* Myb that disrupt its functions *in vitro* and *in vivo* also disrupt these activities in *C. elegans*. We speculate that nematodes and other animals may contain another protein that can bind to LIN9 and LIN52 in order to activate transcription of genes repressed by DREAM complexes.

## Abstract (precis)

**Summary:** Despite nematodes having lost their animal-type Myb proteins 500 million years ago, the *Caenorhabditis elegans* MuvB complex still functionally interacts with the *Drosophila melanogaster* Myb protein *in vitro* and *in vivo*.

## INTRODUCTION

The *Myb* gene family was discovered due to the retroviral transduction of the *c-Myb* proto-oncogene that created the *v-Myb* oncogene of the avian myeloblastosis virus (Lipsick and Wang, 1999). Vertebrate animals including humans have three paralogous *Myb* genes (*A-Myb/MYBL1*, *B-Myb/MYBL2* and *c-Myb/MYB*) (Davidson et al., 2004; Lipsick, 1996). The fruit fly *Drosophila melanogaster* and many other invertebrate species contain a single essential animal-type *Myb* gene that is closely related to vertebrate *B-Myb* (Davidson et al., 2004; Katzen and Bishop, 1996; Katzen et al., 1985; Lipsick, 1996; Manak et al., 2002; Okada et al., 2002). The vertebrate *A-Myb* and *c-Myb* genes appear to have arisen by two rounds of gene duplication and divergence from a *B-Myb*-like ancestral gene (Davidson et al., 2013). Consistent with this model, vertebrate *B-Myb*, but neither *A-Myb* nor *c-Myb*, can complement the cell-cycle defects observed in *Drosophila*-*Myb*-null mutant animals (Davidson et al., 2005; Manak et al., 2002).

Animal Myb-type proteins all contain a broadly conserved pan-eukaryotic amino-terminal DNA-binding domain and animal-specific domains (Davidson et al., 2004). The vertebrate A-Myb and c-Myb proteins also share a central transcriptional activation domain that is not well conserved in vertebrate B-Myb or invertebrate Myb proteins. Surprisingly, the animal-specific carboxy-terminus of *Drosophila* Myb is both necessary and sufficient for rescue of the adult lethality of a *Myb*-null mutant, for proper association with chromatin, for transcriptional activation of essential G2/M phase genes and for mitotic cell cycle progression (Andrejka et al., 2011; Wen et al., 2008). Alanine substitutions of evolutionarily conserved motifs identified a short peptide sequence that is required for all these functions (Andrejka et al., 2011).

Biochemical purification of an activity that bound to DNA near a developmentally regulated origin of replication in a *Drosophila* chorion locus led to the discovery of a multiprotein complex that contains Myb and several Myb-interacting proteins (Beall et al., 2002). Similar complexes called Myb-MuvB, which contain either B-Myb or less frequently A-Myb, were later identified in human cells (Sadasivam and DeCaprio, 2013). In addition to *Drosophila* Myb or vertebrate B-Myb, these complexes contain Mip130/LIN9, Mip120/LIN54, Mip40/LIN37, p55CAF1/RbAp48 and LIN52. These five additional proteins, known as the MuvB core, can also associate with *Drosophila* E2F2, DP and RBF1 or RBF2 or their vertebrate homologs (E2F4 or E2F5, DP1 or DP2, p107 or p130), to form complexes now called DREAM (Sadasivam and DeCaprio, 2013). A large holocomplex containing Myb, E2F, DP and RB family proteins together with the MuvB core was identified in *Drosophila* embryos, but has not been observed in human cell lines (Korenjak et al., 2004; Lewis et al., 2004; Sadasivam and DeCaprio, 2013).

*Myb* loss-of-function mutants in *Drosophila* display mitotic cell-cycle defects and aberrations in ploidy in somatic tissues (DeBruhl et al., 2013; Fung et al., 2002; Katzen et al., 1998; Manak et al., 2002, 2007; Okada et al., 2002). In both *Drosophila* and human cell lines, the Myb-MuvB complex has been shown to activate the transcription of genes essential for G2/M progression in mitotically active cells, whereas the DREAM complex represses these genes (Dimova et al., 2003; Fischer and Müller, 2017; Georlette et al., 2007; Litovchick et al., 2007; Muller et al., 2017; Osterloh et al., 2007; Pilkinton et al., 2007). These complexes have also been implicated in human cancer initiation and progression. For example, a high level of *B-Myb/MYBL2* expression in breast cancer is a clinically useful predictor of tumor recurrence and decreased patient survival (Amatschek et al., 2004; Paik et al., 2004; Thorner et al., 2009). Furthermore, extensive DNA sequencing has revealed that approximately one-half of human breast cancer specimens contain a genetic alteration in at least one of the genes encoding subunits of these two complexes (Fig. S1).

Remarkably, all of the proteins in the Myb-MuvB and DREAM complexes, with the exception of Myb itself, are encoded by homologs of synMuvB group genes in the nematode *Caenorhabditis elegans* (Lipsick, 2004). In brief, dominant gain-of-function mutations in the EGF=>RAS=>RAF=>MEK=>MAPK=>ETS pathway caused a multivulval (Muv) phenotype in *C. elegans* (Sternberg and Han, 1998). Recessive loss-of-function mutations in *lin-8* and *lin-9* together caused a synthetic multivulval phenotype (synMuv) (Horvitz and Sulston, 1980). Additional genetic screens identified two groups of genes (synMuvA and synMuvB) in which any group A mutation could cooperate with any group B mutation to cause this synMuv phenotype (Ferguson and Horvitz, 1989). The proteins encoded by synMuvA and synMuvB genes redundantly repress ectopic expression of the secreted LIN-3/EGF protein that normally controls vulval development via the RAS pathway (Cui et al., 2006; Myers and Greenwald, 2005; Saffer et al., 2011; Sternberg and Han, 1998). The synMuvB genes also regulate transgene silencing, cell cycle progression, repression of germline-specific genes in somatic cells, RNA interference (RNAi), and X chromosome gene expression (Boxem and van den Heuvel, 2002; Hsieh et al., 1999; Petrella et al., 2011; Tabuchi et al., 2014; Wang et al., 2005).

In *Drosophila*, the DREAM complex encoded by homologs of nematode synMuvB genes represses the expression of G2/M phase genes and also represses ectopic expression of the carbon dioxide receptor in olfactory neurons (DeBruhl et al., 2013; Sim et al., 2012; Wen et al., 2008). The *Drosophila* Myb protein is required to relieve this DREAM-mediated repression for mitotic cell-cycle progression and for carbon dioxide receptor expression in the appropriate neurons. *Drosophila* Myb also acts in opposition to the DREAM complex to regulate chorion gene amplification in ovarian follicle cells and programmed neuronal cell death (Beall et al., 2004, 2002; Rovani et al., 2012). Interestingly, recent studies in *C. elegans* have shown that the MuvB complex can effectively repress gene expression in the absence of the LIN-35 RB-family protein that was previously thought to be required for repression by DREAM complexes (Goetsch et al., 2017). Although *C. elegans* and other nematode species contain two *Myb*-related genes that encode homologs of the CDC5/CEF1 splicing factor and the SNAPc small nuclear RNA transcription factor, they do not contain an animal-type *Myb* gene that might relieve repression by DREAM complexes (Davidson et al., 2004).

The animal-specific carboxy-terminus of *Drosophila* Myb is both necessary and sufficient for binding to the MuvB core complex in cell lysates (Andrejka et al., 2011). In addition, two alanine substitution mutants that greatly diminished the biological activities of *Drosophila* Myb *in vivo* also inhibit its binding to the MuvB core complex. Studies with recombinant proteins identified conserved Myb-binding domains of human LIN9 and LIN52 that in concert are sufficient for binding to the homologous MuvB-binding domain of human B-Myb and *Drosophila* Myb (Guiley et al., 2018). Structural determination by X-ray crystallography revealed a coiled-coil comprised of human LIN9 and LIN52 α-helices, which together form a binding site for the MuvB-binding domain of B-Myb. Furthermore, the amino acids in B-Myb homologous to those disrupted by the non-functional *Drosophila* Myb alanine substitution mutants make critical contacts with the LIN9 and LIN52 Myb-binding domains.

Surprisingly, the residues in human LIN9 and LIN52 that contact B-Myb are highly conserved in *C. elegans*, which itself lacks an animal-type Myb protein. Interestingly, in structure-based molecular modeling studies, the homologous peptides of nematode LIN9 and LIN52 could readily accommodate the MuvB-binding domains of animal Myb proteins. We therefore decided to test whether the MuvB-binding domain of *Drosophila* Myb can functionally interact with the putative Myb-binding domains of *C. elegans* LIN9 and LIN52, both *in vitro* and *in vivo*.

## RESULTS

### Evolutionary conservation of animal-type Myb proteins

**Fig. 1.**
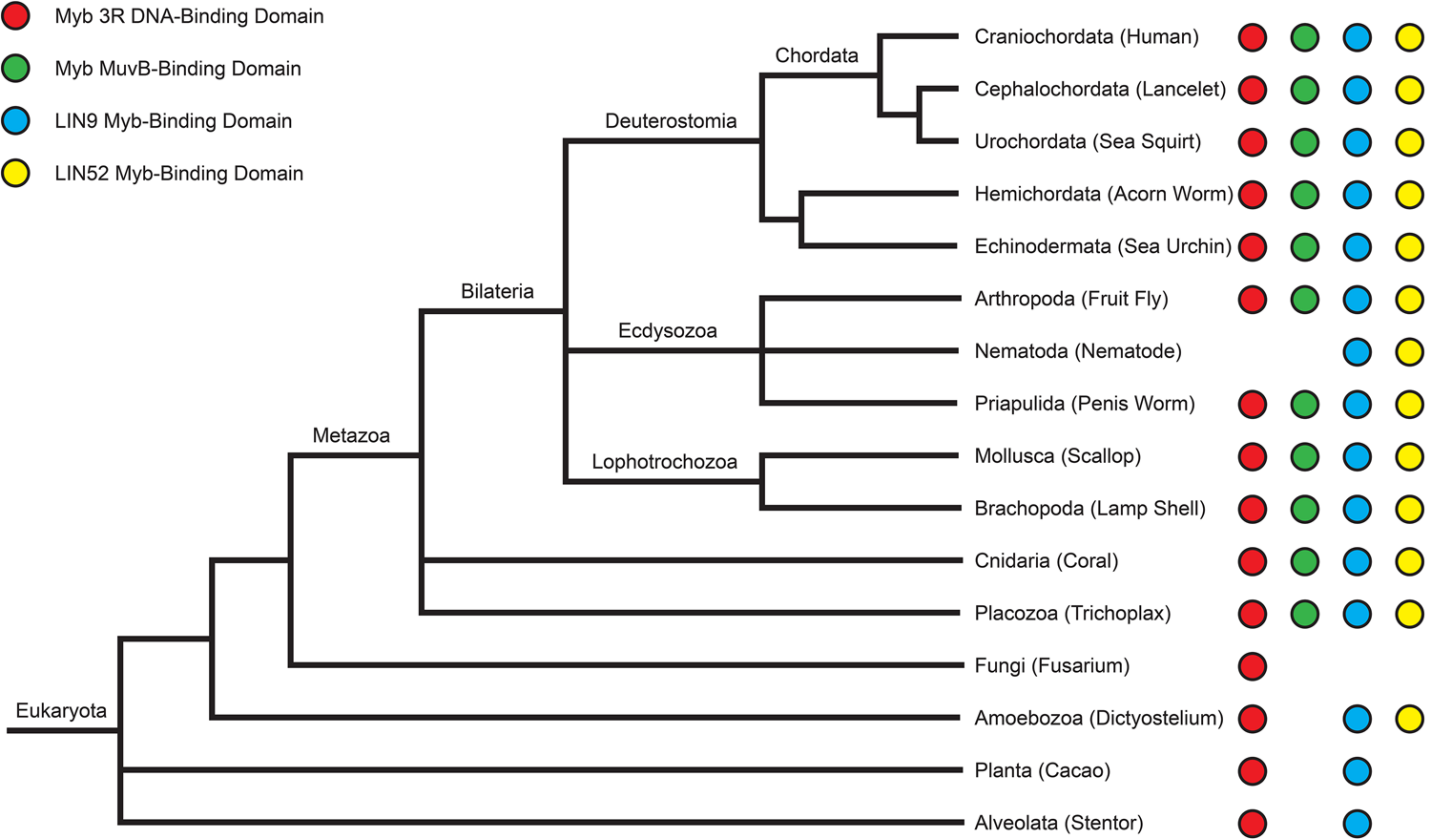
**Evolutionary conservation of Myb three-repeat (3R) DNA-binding domains, MuvB-binding domains of Myb proteins, and Myb-binding domains of LIN9 and LIN52.** A partial phylogenetic tree of the current view of eukaryotic evolution (http://tolweb.org/tree/) shows the presence or absence of the indicated protein domains in representative species from diverse clades: human (*Homo sapiens*), lancelet (*Branchiostoma belcheri*), sea squirt (*Ciona intestinalis*), acorn worm (*Saccoglossus kowalevskii*), sea urchin (*Strongylocentrotus purpuratus*), fruit fly (*D. melanogaster*), nematode (*C. elegans*), penis worm (*Priapulus caudatus*), scallop (*Mizuhopecten yessoensis*), lamp shell (*Lingula anatina*), coral (*Stylophora pistillata*), trichoplax (*Trichoplax adhaerens*), fusarium (*Fusarium sp. AF-4*), dictyostelium (*Dictyostelium discoideum*), cacao (*Theobroma cacao*) and stentor (*Stentor coeruleus*). Displayed branch lengths are unscaled.

**Fig. 2.**
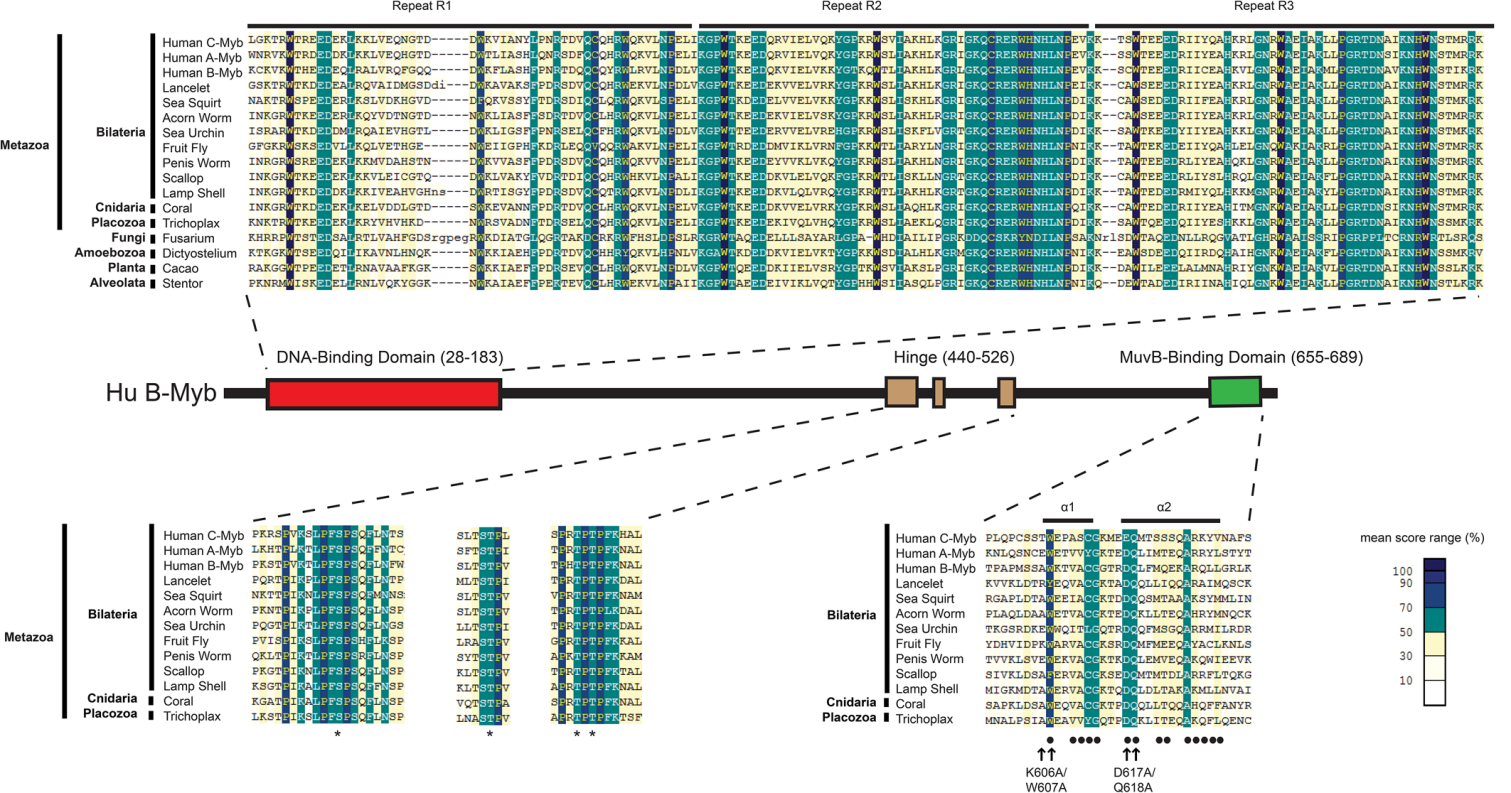
**Sequence alignments of conserved** **animal-type Myb protein domains.** A schematic diagram of the human B-Myb protein shows the relative positions and amino acid sequence numbers of the conserved domains that define animal-type Myb proteins. Local multiple protein sequence alignments were constructed using MACAW with the BLOSUM62 scoring matrix (Schuler et al., 1991). The alignment shading indicates the mean score at each position as shown in the color key. Horizontal bars above the DNA-binding domain alignment indicate three tandem Myb repeats (R1, R2, R3). Asterisks below the hinge domain alignments indicate known Cyclin A-CDK2 phosphorylation sites in the hinge region human B-Myb as described by Werwein et al. (2019). The central hinge domain alignment contains a binding site for the Plk1 polo-family protein kinase. Horizontal bars above the MuvB-binding domain alignment indicate α-helices in the human B-Myb crystal structure with human LIN9 and LIN52 (Guiley et al., 2018). Black dots below the MuvB-binding domain alignment indicate amino acids of human B-Myb that contact human LIN9 or LIN52 in the crystal structure. Arrows below the MuvB-binding domain indicate amino acids substituted by alanine in two *Drosophila* Myb mutants used in experiments in this study (Andrejka et al., 2011).

Searches of public sequence repositories revealed that animal-type Myb proteins, characterized by an amino-terminal DNA-binding domain, composed of three tandem Myb repeats, a central proline-rich ‘hinge’ and a carboxy-terminal MuvB-binding domain, are present in species of all phyla of the superphylum Deuterostomia, including Chordata (human, lancelet, sea squirt), Hemichordata (acorn worm), and Echinodermata (sea urchin) (Figs 1 and 2). Animal-type Myb proteins, defined as having these three domains, are also present in species of widely divergent invertebrate animal phyla including Arthropoda (fruit fly), Priapulida (penis worm), Mollusca (scallop), Brachiopoda (lamp shell), Cnidaria (coral), and Placozoa (*Trichoplax*). Surprisingly, none of the 25 completely sequenced and widely divergent species of Nematoda (round worm) contain an animal-type Myb protein, although they do contain more distant Myb-related proteins of the SNAPc and Cdc5/CEF1 families that are also present in a wide range of eukaryotes including fungi (Davidson et al., 2004). Nematodes have not left a fossil record, but analyses of molecular evolution have led to estimates of between 600 and 1300 million years since their divergence from other animals (Coghlan, 2005).

The phylogenetic relationship of nematodes to chordates and arthropods within the Bilateria has been controversial (Holton and Pisani, 2010; Telford and Copley, 2005). Nevertheless, the presence of animal-type *Myb* genes in species of the non-bilaterian ‘out group’ phyla, Cnidaria (coral) and Placozoa (*Trichoplax adherens*), argues strongly that animal-type *Myb* genes were present in the last common ancestor of all these widely divergent animal species, including nematodes. Therefore, a common ancestor of all modern nematodes appears to have lost its animal-type *Myb* gene. A similar loss may have occurred during the evolution of some other phyla of the Bilateria, but in those cases there are not as many divergent species with completely sequenced genomes as in the Nematoda.

The presence of highly conserved three-repeat Myb DNA-binding domains in species of the kingdoms of Fungi (*Fusarium*) and Plants (*Cacao*) and in the ‘orphan’ clades of Amoebozoa (*Dictyostelium*) and Avleolata (*Stentor*) suggests that a common ancestor of most if not all modern eukaryotes contained this domain (Figs 1 and 2). However, the MuvB-binding domain and adjacent proline-rich hinge that are also present in animal-type Myb proteins have not been found in any of the known Myb-related proteins of widely divergent non-animal species. This result suggests that the MuvB-binding domain and adjacent proline-rich hinge emerged subsequent to the divergence of Metazoa from other eukaryotic kingdoms and ‘orphan’ clades.

### Evolutionary conservation of the Myb-binding domains of LIN9 and LIN52

**Fig. 3.**
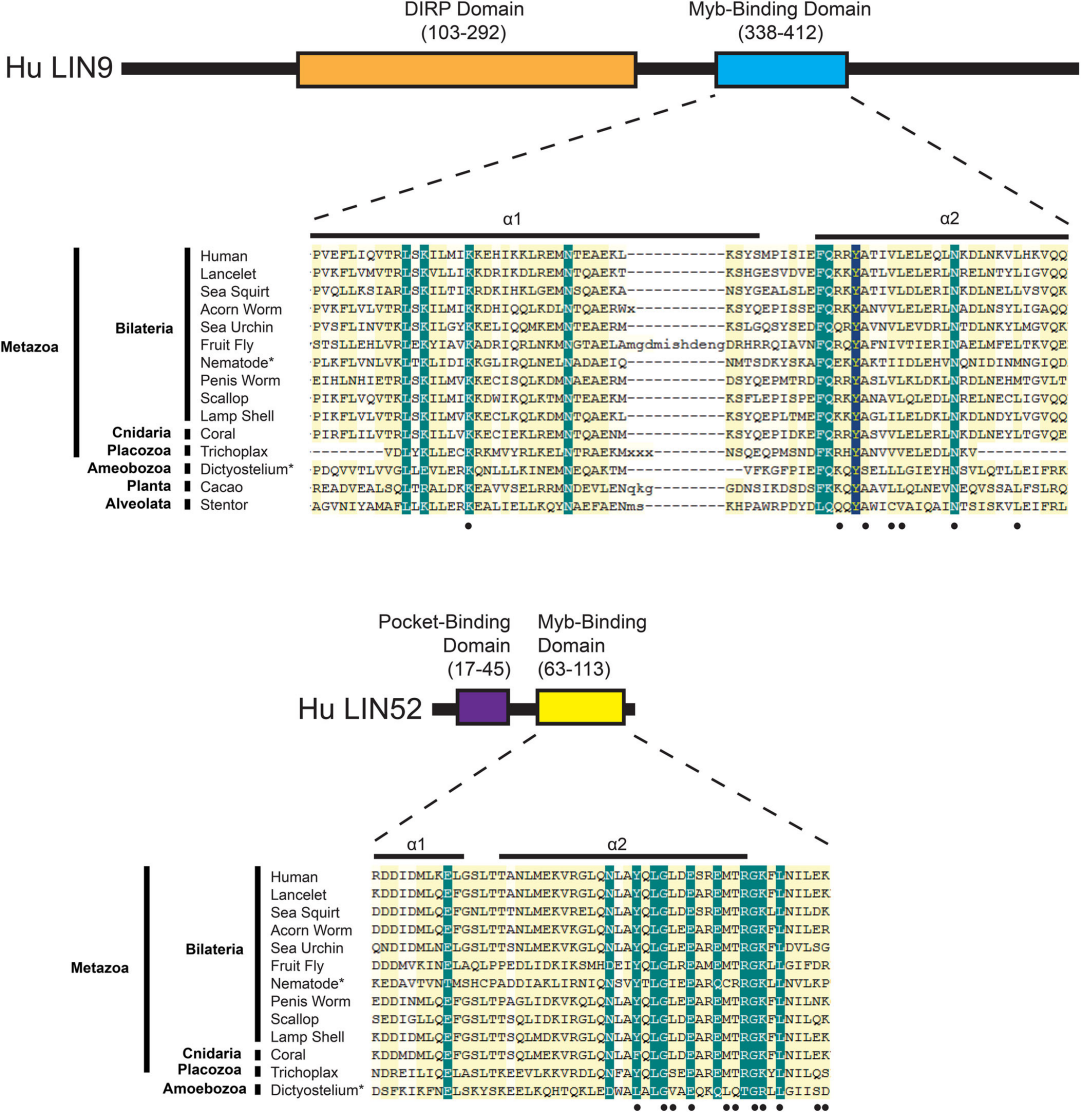
**Sequence alignments of the Myb-binding domains of LIN9 and LIN52.** Schematic diagrams of the human LIN9 and LIN52 proteins show the relative positions and amino acid sequence numbers of the conserved domains. Horizontal bars above the Myb-binding domain alignments indicate α-helices in the crystal structure of human LIN9 and LIN52 bound to human B-Myb (Guiley et al., 2018). Black dots below the Myb-binding domain alignments indicate amino acids that contact human B-Myb in the crystal structure. Alignments are not shown for the DIRP domain of LIN9 (pfam 06584) (White-Cooper et al., 2000), the function of which remains unknown, or for the pocket-binding domain of LIN52 that binds to the human RB-related p107 and p130 proteins but not to human RB itself (Guiley et al., 2015).

Searches of public sequence repositories revealed that the Myb-binding domain of LIN9 proteins is conserved in a wide range of species within the Metazoa, including those in the Nematoda that lack an animal-type Myb protein (Figs 1 and 3). Sequences homologous to the Myb-binding domain of LIN9 were also identified in species within the Planta, Amoebozoa and Alveolata that have proteins containing an animal-type Myb DNA-binding domain but lacking an MuvB-binding domain. The deep evolutionary conservation of the Myb-binding domain of LIN9 in the absence of the MuvB-binding domain of Myb suggests that the former domain is likely to have another function. The lack of any proteins homologous to either the DIRP (domain in Rb-related pathway, Pfam 06584) (White-Cooper et al., 2000) or Myb-binding domains of LIN9 in Fungi suggests that a common ancestor of the modern fungal species lost its *LIN9* gene after its divergence from the other kingdoms of eukaryotes. In addition, the presence of LIN9-related proteins with a DIRP domain but no Myb-binding domain in some plant species including the intensively studied thale cress (*Arabidopsis thaliana*) suggests that these domains can function independently of one another.

Proteins of the LIN52 family are not as evolutionarily widespread as those of the LIN9 family and were only identified in species within the Metazoa and Amoebozoa (Figs 1 and 3). These LIN52 homologs contain both a pocket-binding domain and an Myb-binding domain. The former is consistent with the presence of RB ‘pocket’ family proteins in these species. The absence of Myb family MuvB-binding domains in Nematoda and Amoebozoa, despite the presence of both LIN9 and LIN52 Myb-binding domains, again suggests that these domains have an additional evolutionarily conserved function.

### The MuvB-binding domain of *Drosophila* Myb can bind to a nematode LIN9-LIN52

**Fig. 4.**
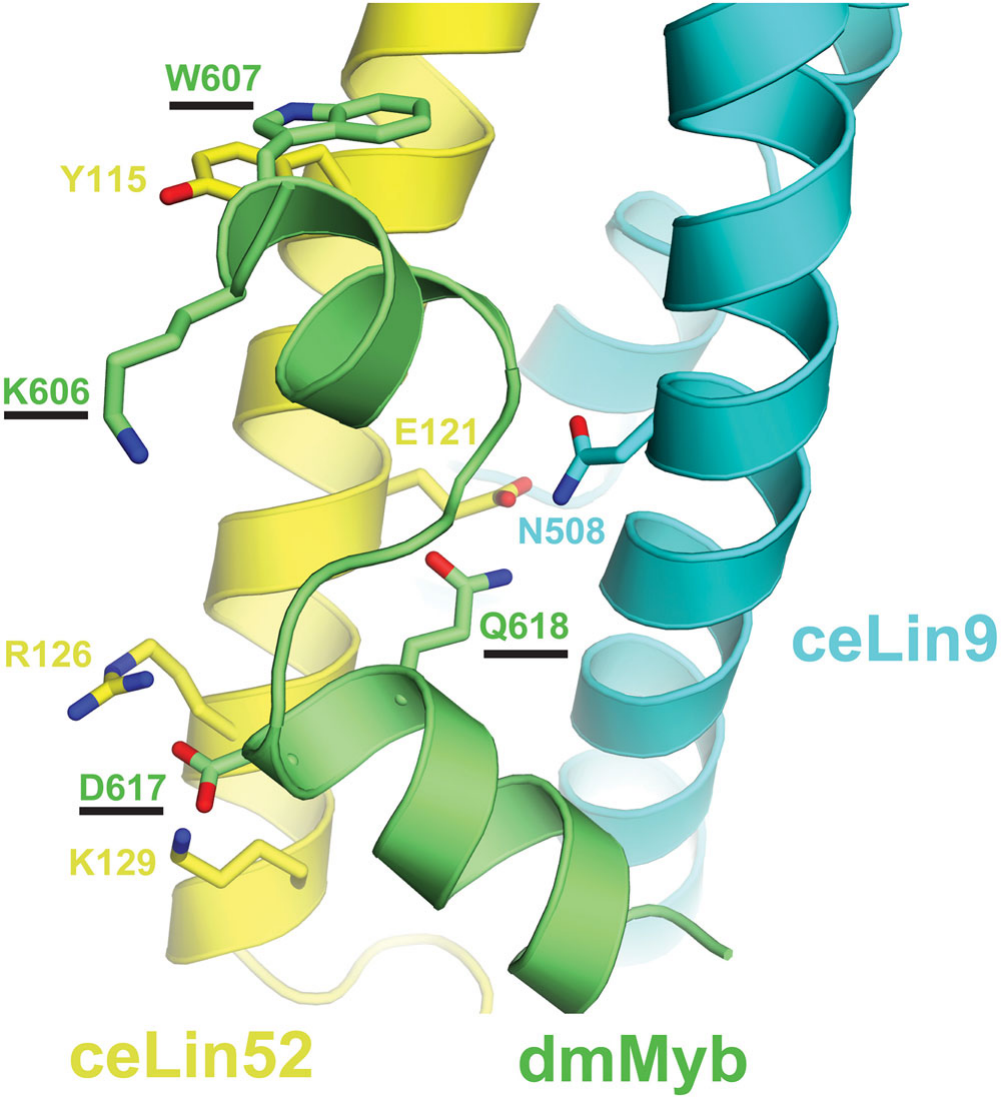
**Structural modeling of the *Drosophila* Myb MuvB-binding domain bound to the Myb-binding domains of nematode LIN9 and LIN52.** Protein sequences of *Drosophila* Myb (dmMyb; green), *C. elegans* LIN9 (ceLIN9; cyan) and *C. elegans* LIN52 (ceLIN52; yellow) were modeled into the human crystal structure (PDB ID: 6C48) using MODELLER (Webb and Sali, 2017). Amino acid numbering shown is for these *Drosophila* and *C. elegans* proteins. The four amino acids substituted by alanine in the two *Drosophila* Myb mutants used in experiments in this study are underlined.

Although no sequenced species of Nematoda contains an animal-type Myb protein, they nevertheless do contain conserved Myb-binding domains in their LIN9 and LIN52 family proteins (Figs 1 and 3). Furthermore, the amino acids in the human LIN9 and LIN52 proteins that make direct contacts with human B-Myb in a structure determined by X-ray crystallography are well conserved in the LIN9 and LIN52 proteins of the intensively studied nematode *C. elegans* (Guiley et al., 2018) (Fig. 3). Molecular modeling based on this structure predicts that the *C. elegans* LIN9 and LIN52 proteins are likely to be capable of binding to the MuvB-binding domain of *Drosophila* Myb (Fig. 4). Furthermore, conserved amino acids in *Drosophila* Myb (K606, W607, D617, Q618) that were previously shown to mediate biochemical interactions with the *Drosophila* MuvB core complex *in vitro* and to be required for the function of *Drosophila* Myb *in vivo* are predicted to contact *C. elegans* LIN9 and LIN52 (Andrejka et al., 2011). The homologous amino acids in human B-Myb were shown to contact human LIN9 and LIN52 in the crystallographic structure (Guiley et al., 2018).

**Fig. 5.**
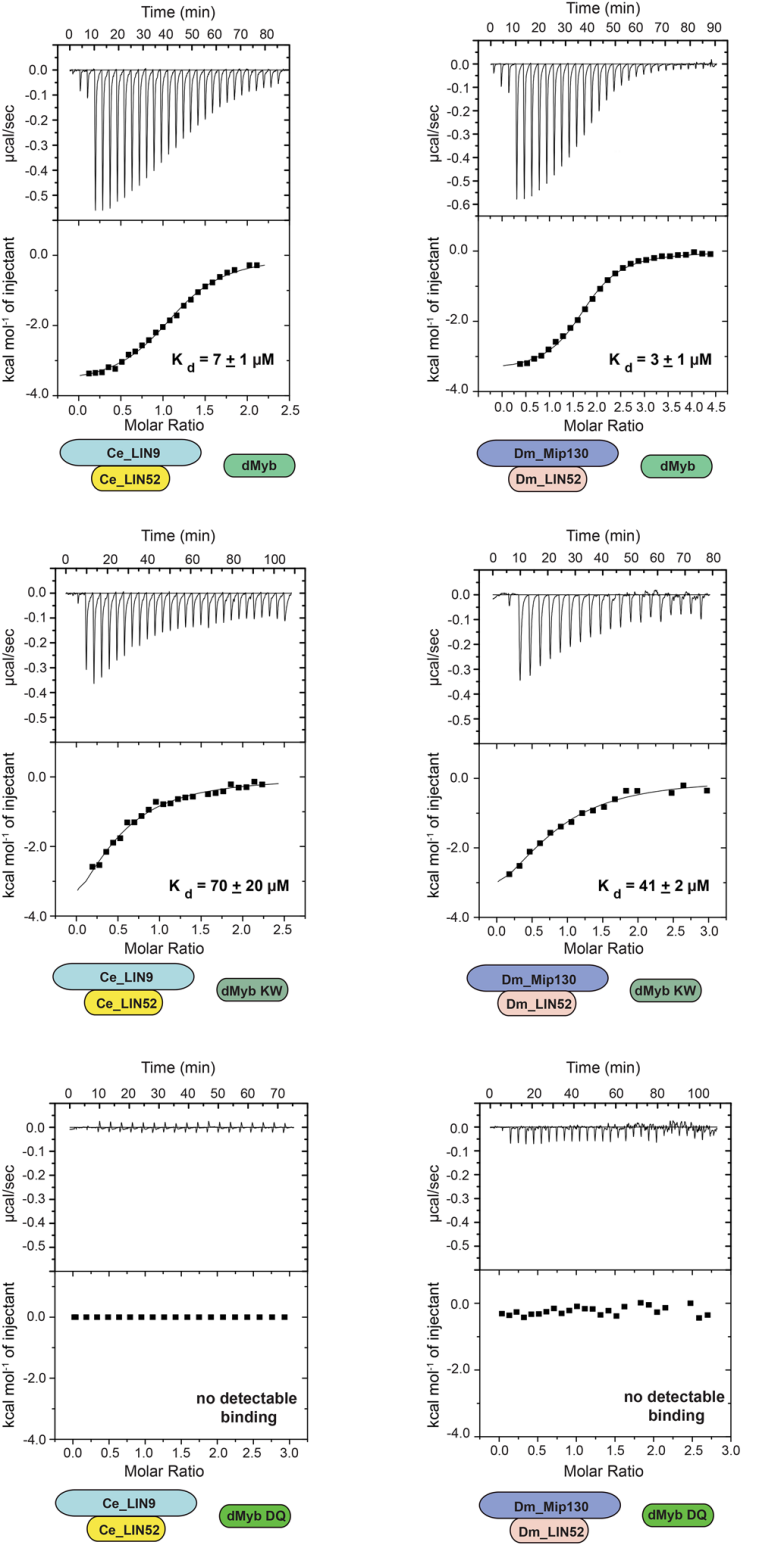
**The Myb-binding domains of *C. elegans* LIN9 and LIN52 bind the *Drosophila* MuvB-binding domain of *Drosophila* Myb *in vitro*.** Recombinant *C. elegans* LIN9-LIN52 heterodimeric Myb-binding domains produced in *E. coli* were purified and then assayed for binding to recombinant *Drosophila* Myb MuvB-binding domain, using isothermal titration calorimetry. Each panel displays a representative experiment using the proteins diagrammed below the panel. The raw data are presented above and the fitted binding curve is presented below. The mean calculated K_d_ values and standard deviations from three replicate experiments are shown below each panel.

To test whether the conservation of Myb-binding domain sequences in *C. elegans* LIN9 and LIN52 results in conservation of protein function, the relevant recombinant protein domains were produced in *Escherichia coli* and purified using affinity, ion-exchange, and size-exclusion chromatography (Fig. S2). Reconstituted heterodimeric LIN9-LIN52 Myb-binding domain complexes were tested for their ability to bind the MuvB-binding domain of *Drosophila* Myb using isothermal titration calorimetry (Fig. 5). The *C. elegans* and the *Drosophila* LIN9-LIN52 heterodimers bound to *Drosophila* Myb with very similar affinities (K_d_=7 or 3 μM, respectively). The K606A/W607A double-substitution mutant of *Drosophila* Myb caused an approximately ten-fold reduction of binding to either *C. elegans* or *Drosophila* LIN9-LIN52 (K_d_=70 or 41 μM, respectively). Furthermore, the D617A/Q618A double substitution mutant of *Drosophila* Myb abolished any detectable binding to either *C. elegans* or *Drosophila* LIN9-LIN52. These experiments show that despite over 500 million years since nematodes appear to have lost their animal-type Myb genes, their LIN9 and LIN52 proteins are still capable of binding to the MuvB-binding domain of *Drosophila* Myb in a similar fashion to *Drosophila* LIN9 and LIN52.

### Expression of *Drosophila* Myb in *C. elegans* causes a synthetic multivulval phenotype

The *lin-9* gene was discovered in *C. elegans* because a loss-of-function mutation cooperated with a second loss-of-function mutation in *lin-8* to cause a synthetic multivulval (synMuv) phenotype (Horvitz and Sulston, 1980). Additional genetic screens identified a group of genes (class A synMuv) for which a loss-of-function mutant could cooperate with a *lin-9* mutant to cause a synMuv phenotype. A second group of genes (class B synMuv) including both *lin-9* and *lin-52* were identified, for which a loss-of-function mutant could cooperate with a class A synMuv loss-of-function mutant to cause a synMuv phenotype (Ferguson and Horvitz, 1989). The *Drosophila* Myb protein can cause the transcriptional activation of genes *in vivo* that are repressed by homologs of proteins encoded by synMuvB genes including Mip130/LIN9, E2F2, RBF1 and RBF2 (DeBruhl et al., 2013; Sim et al., 2012; Wen et al., 2008). Therefore, we hypothesized that expression of *Drosophila* Myb in *C. elegans* might cause a synMuv phenotype similar to that seen in loss-of-function mutants of the endogenous synMuvB genes.

To test this hypothesis, GFP::Myb fusion proteins or a GFP-only (GFP) control protein were expressed in *C. elegans* under control of a heat-shock promoter using stably integrated single-copy transgenes. This was accomplished using the CRISPR-Cas9 system to promote transgene integration at a specific site on chromosome II (Dickinson et al., 2013; Frøkjaer-Jensen et al., 2008). Four different GFP::Myb fusion proteins were expressed in this manner: full-length wild type (Myb), a mutant of Myb lacking its N-terminal DNA-binding domain but containing its C-terminal MuvB-binding domain (C-term), the K606A/W607A (KW) double-substitution mutant and the D617A/Q618A (DQ) double-substitution mutant. Homozygous transgenic strains were genotyped by PCR of genomic DNA (Fig. S3). Following heat shock of transgenic worms, similar levels of nuclear GFP fluorescence were observed in gut cells in the majority of animals examined on a dissection microscope. Detailed examination of live worms on a spinning disc confocal microscope, with a 40X objective, revealed nuclear GFP in the vulval precursor cells and adjacent hypodermal cells. Transgene expression in these cells varied from animal to animal, and from cell to cell within individual animals (Fig. S4). We did not observe misexpression of a germline-specific *pgl-1::RFP* reporter in somatic cells following pulses of Myb expression under control of the *hsp-16* promoter (Fig. S4). However, this promoter may not function sufficiently early during development to turn on germline genes in somatic cells (Stringham et al., 1992). It is also possible that sustained rather than transient expression of Myb in a somatic tissue would be required to mimic this phenotype of endogenous synMuvB loss-of-function mutants (Petrella et al., 2011; Wang et al., 2005).

**Fig. 6.**
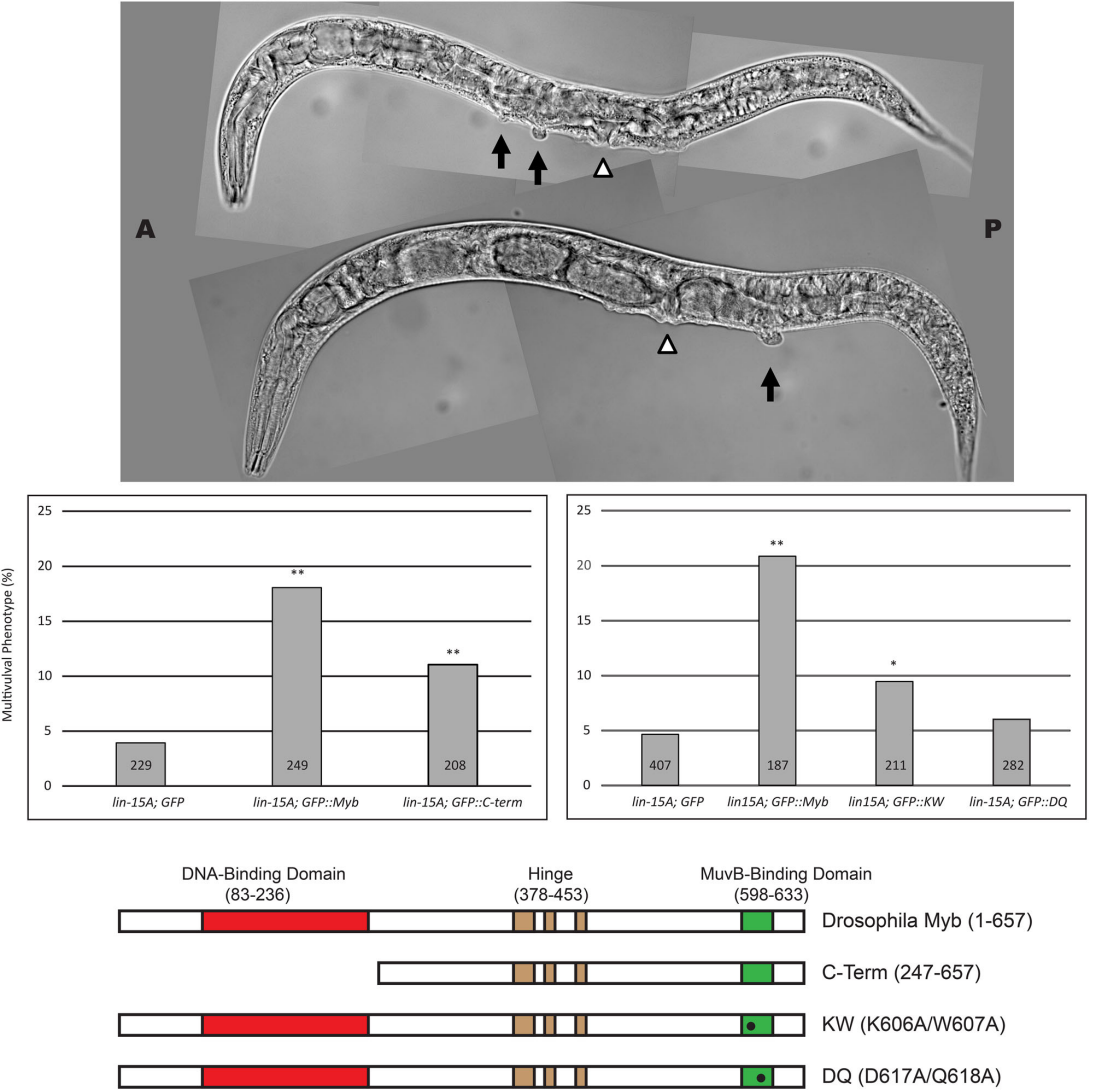
***Drosophila* Myb causes a synthetic multivulval phenotype in *C. elegans*.** Top panel: the indicated strains were heat-shocked as L2/L3 larvae, then scored as adults for the presence of a multivulval phenotype in a *lin-15A* mutant background. DIC images of two representative multivulval worms of the *lin-15A; GFP::Myb* genotype are shown (open arrowheads indicate the normal vulval opening, black arrows indicate ectopic vulvae). Middle panel: histograms show the incidence of multivulval worms in two different experiments using strains of the indicated genotypes. Statistical significance relative to the *lin-15A; GFP* control strain was determined using a two-tailed Z-test. One asterisk indicates a significance of 0.05 or less; two asterisks indicate a significance of 0.01 or less. Numbers within the bars indicate total number of worms scored for the indicated genotype. Bottom panel: schematic diagrams of the *Drosophila* Myb wild-type and mutant proteins expressed in transgenic worms. All of the Myb proteins contained a GFP tag fused at their amino termini (not shown).

The transgenes were each crossed into a strong synMuvA (*lin-15A*) loss-of-function mutant background and doubly homozygous strains were isolated. These new strains were examined for the adult synMuv phenotype following induction of transgenic GFP or GFP::Myb protein expression by a single 15-min heat shock during the L2/L3 stages of larval development. Consistent with a previous report of temperature dependence, the *lin-15A* allele we used caused a Muv phenotype in approximately 5% of worms following heat shock with or without the *GFP* control transgene (Saffer et al., 2011). In contrast, induction of the full-length wild-type *GFP::Myb* transgene in the same *lin-15A* mutant background elevated the incidence of a synMuv phenotype to approximately 20% of worms (Fig. 6).

A mutant *Drosophila* Myb protein (C-term) lacking its highly conserved DNA-binding domain was previously shown to physically interact with the *Drosophila* MuvB core complex (Andrejka et al., 2011). In *Myb*-null mutant flies, this C-term Myb mutant protein localized to the cell nucleus, localized to chromatin, activated the expression of G2/M phase genes and rescued G2/M-phase cell cycle progression. Furthermore, the C-term Myb mutant protein rescued adult viability approximately 70% as well as wild-type Myb, but only at low temperatures (Andrejka et al., 2011; Wen et al., 2008). The C-term *Drosophila* Myb mutant protein also caused an elevated incidence of the synMuv phenotype when in combination with a *lin-15A* mutation, albeit less efficiently (approximately 10% incidence) than the full-length Myb protein (approximately 20% incidence) (Fig. 6). Neither *Drosophila* Myb protein caused a multivulval phenotype in worms that were wild type for *lin-15A*. These results show that the *Drosophila* Myb protein in combination with loss of *lin-15A* causes a synMuv phenotype in *C. elegans*, which itself has no animal-type Myb gene or protein of its own. Furthermore, as was previously observed in *Drosophila*, the highly conserved Myb DNA-binding domain is not required for this synMuv phenotype in *C. elegans*.

The KW and DQ double-alanine-substitution mutants of *Drosophila* Myb have previously been shown to abolish detectable immunoprecipitation of the *Drosophila* MuvB core complex from cell extracts *in vitro*, and to be greatly diminished in rescuing expression of G2/M phase genes, cell cycle progression and adult viability *in vivo* in *Myb*-null mutant flies (Andrejka et al., 2011). Similar mutants of the human B-Myb protein were subsequently shown to inhibit its physical interaction with the human LIN9-LIN52 Myb-binding domain complex (Guiley et al., 2018). The KW mutant of *Drosophila* Myb resembled the C-terminal Myb transgene in causing an approximately 10% incidence of the synMuv phenotype when in combination with a *lin-15A* mutant *Drosophila* (Fig. 6). The DQ mutant of *Drosophila* Myb did not cause an elevation in the incidence of a synMuv phenotype in a *lin-15A*-mutant background relative to the controls (all approximately 5%).

The differing abilities of the KW and DQ mutants of *Drosophila* Myb to cause a synMuv phenotype in *C. elegans* correlate with their abilities to bind either weakly or not at all to the *C. elegans* LIN9-LIN52 Myb-binding domain complex *in vitro* (Fig. 5). The relative activities of these mutant proteins in nematodes also correlate with the ability to rescue the adult viability of *Drosophila Myb*-null mutants at low temperature under control of the *Myb* promoter: the KW mutant weakly rescues, while the DQ mutant does not rescue (Andrejka et al., 2011). Taken together, these genetic and biochemical results with mutant Myb proteins suggest that the *C. elegans* LIN9 and LIN52 Myb-binding domains interact with the MuvB-binding domain of *Drosophila* Myb in a fashion very similar to that predicted by evolutionary conservation of protein sequences (Figs 2 and 3) and by structural homology modeling (Fig. 4).

## DISCUSSION

**Fig. 7.**
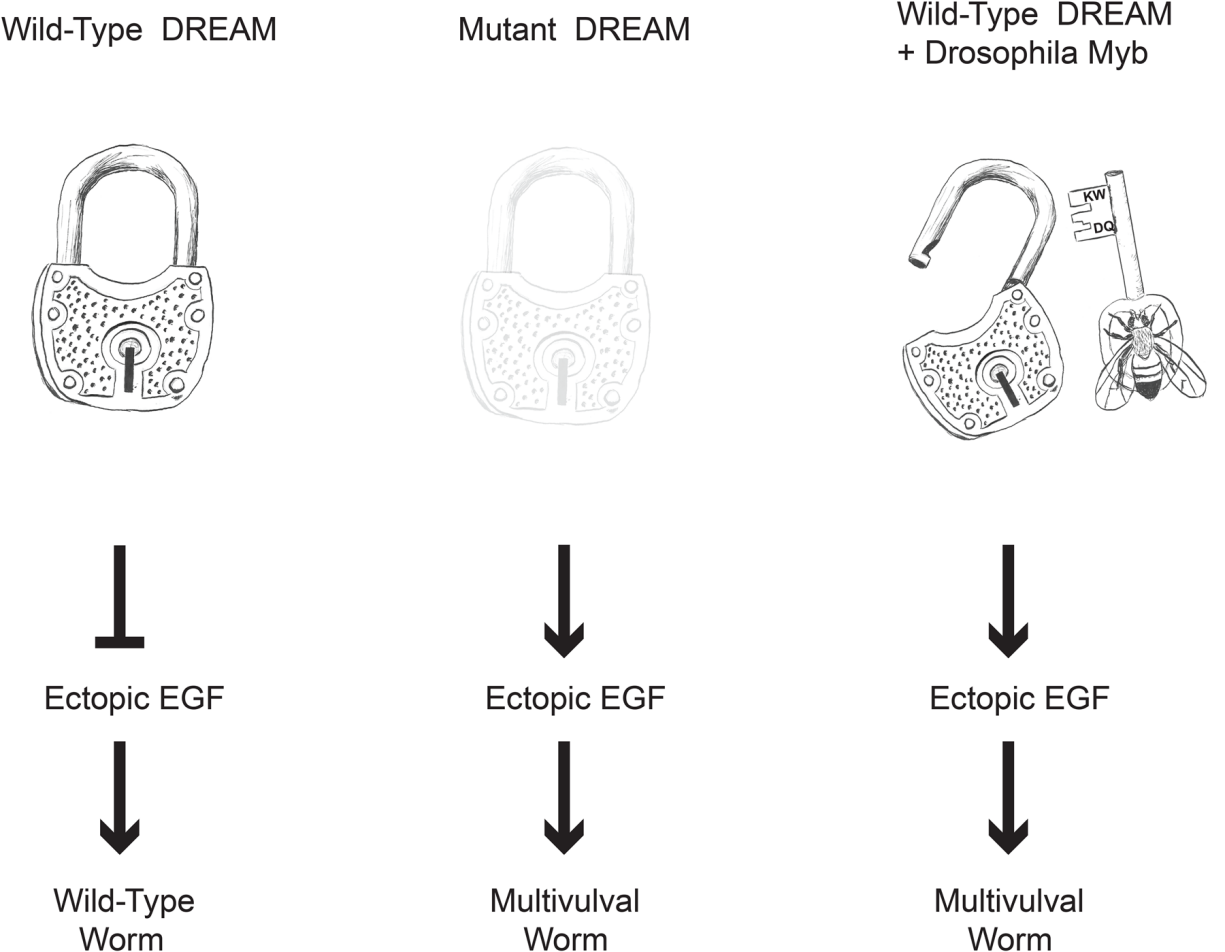
**Model for the mechanism of action of *Drosophila* Myb in *C. elegans*.** The wild-type DREAM complex, which includes LIN9 and LIN52 and other synMuvB proteins, redundantly represses the ectopic expression of LIN3/EGF, resulting in a wild-type worm even in a *lin-15A* synMuvA mutant background. Loss-of-function mutants of synMuvB genes fail to repress ectopic expression of LIN3/EGF, resulting in a synthetic multivulal worm in a *lin-15A* mutant background. Ectopic expression of *Drosophila* Myb overrides repression by the wild-type DREAM complex, causing a synthetic multivulval worm in a *lin-15A* mutant background, presumably due to ectopic expression of LIN-3/EGF. It remains unknown whether nematodes and other animals have a second ‘key’ that can also open the highly conserved DREAM complex ‘lock’.

The MuvB-binding domain of animal-type Myb proteins and the Myb-binding domains of the MuvB subunits LIN9 and LIN52 are conserved in diverse clades of modern Metazoa. Nematodes appear to have lost their animal-type Myb genes and proteins after their divergence from other clades of modern Metazoa (Figs 1 and 2). Nevertheless, the Myb-binding domains of nematode LIN9 and LIN52 have been highly conserved over more than 500 million years in the absence of Myb (Figs 1 and 3). Remarkably, the LIN9-LIN52 Myb-binding domain of *C. elegans* can still bind the MuvB-binding domain of *Drosophila* Myb *in vitro* with a similar affinity and discrimination between mutants as the homologous LIN9-LIN52 domain of *Drosophila* (Figs 4 and 5). Furthermore, the reintroduction of *Drosophila* Myb into *C. elegans* in a synMuvA-mutant background caused a synthetic multivulval phenotype similar to that caused by synMuvB mutants (Fig. 6). These results imply that Myb can act in opposition to transcriptional repression by DREAM-related complexes in *C. elegans*, just as it does in *Drosophila* and in human cell lines (Fig. 7) (Fischer and Müller, 2017; Litovchick et al., 2007; Wen et al., 2008). Our results do not distinguish among models in which *Drosophila* Myb functions as a transcriptional activator in *C. elegans*, as an inhibitor of repression by the MuvB and DREAM complexes, or has both of these activities.

The conservation in *C. elegans* of the amino acids in LIN9 and LIN52 that contact Myb in the crystal structure of their human homologs suggests that there may be another, as-yet-unknown protein in nematodes (and perhaps other species) that can bind to the same LIN9-LIN52 structure in order to activate genes that are repressed by MuvB and DREAM complexes (Fig. 7). Furthermore, the ability of *C. elegans* LIN9 and LIN52 to bind *Drosophila* Myb with a similar affinity and to discriminate among Myb mutants in a fashion similar to *Drosophila* LIN9 and LIN52 suggest a strong selective pressure during the evolution of the Myb-less Nematodes to retain the amino acids that directly contact Myb in humans and *Drosophila*.

It is possible that the amino acids in LIN9 and LIN52 that contact Myb are also essential for the structural integrity of these proteins, thus providing another explanation for their evolutionary conservation. In addition, the LIN9-LIN52 heterodimerization interface may be highly conserved because it is essential for incorporation of these proteins into the MuvB complex (Guiley et al., 2018). On the other hand, the presence of a conserved LIN9 Myb-binding domain in species that have neither an animal-type Myb protein nor a LIN52 protein suggests that this domain of LIN9 may also interact directly with other proteins (Figs 1 and 3).

Genes encoding components of the Myb-MuvB and DREAM complexes are frequently altered in human cancer. For example, 47% of a series of 2051 primary breast cancers were found to contain mutations in one of more of these genes (Fig. S1). Although the *MYBL2* gene encoding the B-Myb protein is altered in only 4% of breast cancers, increased levels of expression of this gene occur more frequently, particularly in basal-like and triple-negative (ER-, PR-, HER2-) breast cancers that generally have a poor prognosis (Amatschek et al., 2004; Thorner et al., 2009). Indeed, *MYBL2* is one of a small number of genes included in the Oncotype DX gene expression test that is widely used to predict clinical outcomes and plan treatment for patients with breast cancer (Bhutiani et al., 2019; Paik et al., 2004).

The Myb-binding domains of LIN9 and LIN52 have been conserved remarkably well during the evolution of animals, even in species like *C. elegans* that lack an animal-type Myb protein. Therefore, it might be difficult for cancer cells to develop resistance to therapeutic drugs that target this site as a treatment for patients whose tumors have elevated levels of B-Myb protein. Furthermore, the conservation of a functional interaction of Myb and LIN9-LIN52 family proteins *in vivo* argues that inexpensive, genetically tractable model organisms such as flies and worms may be useful for screening for biological activity following the identification of lead compounds that inhibit binding *in vitro*.

Our studies highlight the strengths and weaknesses of using evolutionary conservation of primary sequences to predict protein function *in vitro* and *in vivo*. Database searching followed by local alignments of protein sequences permitted the identification of homologs containing conserved Myb-binding and MuvB-binding domains in highly divergent eukaryotic species (Figs 1–3). These data in turn led to inferences about selective pressures for retention of these domains during long periods of evolution. These data also led to direct tests of whether a long lost ‘key’ (the animal-type Myb protein of *Drosophila*) is capable of opening an ancient ‘lock’ *in vivo* (the DREAM complex of *C. elegans*) despite over 500 million years of evolution in the absence of this key (Fig. 7). The high degree of sequence conservation in the MuvB-binding domains of vertebrate c-Myb proteins also led to the prediction that this domain would bind to the Myb-binding domain of LIN9-LIN52. However, this prediction was not borne out (Guiley et al., 2018). This surprising result highlights the need to test hypotheses based on molecular evolution analyses by direct experimentation.

The MuvB-binding domain of vertebrate c-Myb proteins may have been conserved for another function common to all animal-type Myb proteins. Previous studies have provided evidence for negative auto-regulation of animal-type Myb proteins (Ansieau et al., 1997; Dash et al., 1996; Dubendorff and Lipsick, 1999; Dubendorff et al., 1992; Lane et al., 1997; Ramsay et al., 1991; Sakura et al., 1989). Phosphorylation of B-Myb in and around the proline-rich hinge region by the cyclin A-CDK2 protein kinase (Fig. 2) has been shown to cause a conformational change mediated by a peptidyl-prolyl isomerase that can also interact with the c-Myb protein (Leverson and Ness, 1998; Werwein et al., 2019). These results suggest that in addition to interacting with LIN9 and LIN52, the conserved C-terminal MuvB-binding domain may also interact directly or indirectly with the N-terminal DNA-binding domain within animal-type Myb proteins. This could provide a means to regulate the activity of these proteins via phosphorylation and isomerization of their conserved central proline-rich hinge region (Figs 1 and 2). Such a mechanism could explain the selective pressure for retaining a conserved MuvB-binding domain in c-Myb proteins that cannot bind to LIN9 and LIN52.

## MATERIALS AND METHODS

### Database searching and sequence alignment

Homologous protein sequences were identified by BLASTp or tBLASTn searches of the non-redundant protein or nucleotide sequence databases at NCBI as of August 2019 (https://blast.ncbi.nlm.nih.gov/Blast.cgi) for clades of interest based on the phylogenetic trees at the Tree of Life Web Project (http://tolweb.org/tree/). Full-length human or *Drosophila* proteins were used as query sequences. Search results with the lowest E-values (using a cut-off of <1e–10 and the BLOSUM62 scoring matrix) were then ‘back’ BLASTed against the non-redundant human or *Drosophila* protein databases to identify putative orthologs of the query sequences. For *Trichoplax adherens*, all genomic DNA assemblies available at ENSEMBL, some of which were not yet in the NCBI database, were also searched (https://metazoa.ensembl.org/Trichoplax_adhaerens/Info/Annotation/). Conserved domains were identified in homologous protein families and aligned using the MACAW local alignment tool with the BLOSUM62 scoring matrix (Schuler et al., 1991). Accession numbers for all sequences used in these alignments are provided in the Supplemental Information.

### Recombinant protein production and binding assays

LIN9 and LIN52 Myb-binding domains were co-expressed in *E. coli* (Guiley et al., 2018). The open reading frames were synthesized as gBLOCK cassettes (IDT, Coralville, IA, USA), cloned into the following plasmids, then verified by DNA sequencing. LIN9 (*C. elegans* residues 442–559 or *D. melanogaster* residues 571–699) was expressed from a pRSF plasmid without an affinity tag. LIN52 (*C. elegans* residues 75–139 or *D. melanogaster* residues 88–152) was expressed from an engineered pGEX plasmid with an N-terminal GST tag and a TEV protease cleavage site. Proteins were expressed overnight by addition of 1 mM IPTG at 20°C. The heterodimer complex was first purified using glutathione-sepharose affinity chromatography in a buffer containing 40 mM Tris, 150 mM NaCl and 1 mM DTT (pH 8.0). Following elution in buffer containing 10 mM glutathione, the heterodimer was further purified with anion exchange chromatography, cleaved with TEV at 4°C overnight, and the sample was then re-passed over glutathione-sepharose resin to remove the free GST. Final purification was achieved through Superdex 200 size-exclusion chromatography. Wild-type or mutant *D. melanogaster* Myb (residues 602–632) was expressed from the pGEX vector and purified as described for the LIN9-LIN52 complex.

For isothermal titration calorimetry experiments, proteins were concentrated as needed following purification and dialyzed overnight in the same buffer containing 20 mM Tris, 150 mM NaCl and 1 mM beta mercaptoethanol (pH 8.0). The Myb fragment (∼500 μM) was loaded into the MicroCal VP-ITC calorimeter syringe and injected into LIN9-LIN52 heterodimer (∼25–50 μM) at 25°C. Experiments were done in triplicate and data analyzed using the Origin ITC software package.

### Transgenic nematode production

Plasmids containing transgenes for expression in *C. elegans* were constructed using the Gateway system to join four different elements (Merritt and Seydoux, 2010). The 5′ entry plasmid containing the *C. elegans hsp-16* promoter was pCM1.56. The middle entry plasmid containing a *GFP* open reading frame with *C. elegans* introns was pCM1.53. The opening reading frames encoding wild-type, C-term, K606A/W607A or D617A/Q618A mutant *D. melanogaster* Myb were each cloned in-frame into pCM1.53 (Andrejka et al., 2011). The 3′ entry plasmid containing the 3′ UTR of *C. elegans tbb-2* was pCM1.36. The destination vector was CFJ150, which contains an *unc-119* rescue fragment and the genomic DNA sequences flanking a *Mos1* transposon insertion on chromosome II. The final plasmids were validated by DNA sequencing. Site-specific integration of transgenes in the host strain SS1057 with the genotype *unc-119(ed9) III* was accomplished with the CRISPR-Cas9 system (Dickinson et al., 2013; Frøkjaer-Jensen et al., 2008). The single guide RNA with sequence GATATCAGTCTGTTTCGTAA targeting chromosome II near the ttTi5605 *Mos1* insertion site was cloned into pJW1219 using Q5 Site-Directed Mutagenesis (NEB, Ipswich, MA, USA). Worms containing unintegrated transgenic DNA arrays were eliminated by screening for the absence of plasmids pCFJ90 (*Pmyo-2::mCherry*) and pCFJ104 (*Pmyo-3::mCherry*) expressing mCherry in the pharynx and body wall, respectively. Insertion sites were verified by PCR of genomic DNA (Fig. S3).

Strains established from individual hermaphrodites were maintained as homozygotes. The expression of GFP or GFP::Myb fusion proteins by integrated transgenes was verified by fluorescence microscopy 4 h after a 15 min heat shock at 37°C. Each transgene was then crossed into a *lin15-A(n767)* background. Homozygous strains were again established from individual hermaphrodites and verified by PCR of genomic DNA (Fig. S3). Each transgene was also crossed into a *pgl1-1::RFP* reporter background and homozygous strains were established (Fig. S4) (Marnik et al., 2019).

### Phenotypic analysis of nematodes

Young adult hermaphrodites were incubated at 22°C on 10 cm NGM plates with an OP50 bacterial lawn. After depositing embryos for 24 h, adults were removed with an aspirator. Approximately 24 h later a mixed population of L2 and L3 larvae were subjected to a 15 min heat shock in a 37°C water bath. Plates were then incubated for approximately 48 h until all worms were adults. Worms were collected in S buffer (100 mM sodium chloride, 50 mM potassium phosphate, pH 6.0), pelleted by centrifugation, fixed in a solution of 4% paraformaldehyde in phosphate-buffered saline (pH 7.4) for 10 min at 22°C, washed three times in S buffer and then kept on ice prior to scoring. The presence or absence of multiple vulvae was scored by light microscopy after worms were moved to a thin agar slab on a glass slide and then flattened with a glass coverslip.

Tests for inappropriate expression of a germline gene in somatic cells were conducted in a similar fashion in worms containing both an integrated *pgl-1::RFP* reporter gene and the *hsp-16::GFP* or *hsp-16::GFP::Myb* transgene of interest. Following heat shock at 37°C for 20 min, recovery for 20 min at 22°C and a second heat shock at 37°C for 20 min, worms were cultured for an additional 3 h at 22°C. They were then examined for RFP and GFP fluorescence by live imaging with a Yokogawa CSUX-1 spinning disk scanner, a Nikon TE2000-E inverted microscope, a Hamamatsu ImageEM X2 camera, solid state excitation lasers (at 488 nm and 561 nm) and 500–550 nm and 573–613 nm emission filters (Fig. S4).

## Supplementary Material

Supplementary information
