# Supplementary material for: A long lost key opens an ancient lock: Drosophila Myb causes a synthetic multivulval phenotype in nematodes
Source: Biol Open. 2020 May 4;9(5):bio051508. doi: 10.1242/bio.051508 (PMC7225089; doi:10.1242/bio.051508)
Supplement: Supplementary information [file biolopen-9-051508-s1.pdf]

SUPPLEMENTAL INFORMATION

Accession Numbers for Sequences Used in Alignments

Genus species	3R Myb	LIN9	LIN52
Homo sapiens	NP_001123645.1<br>NP_001073885.1<br>NP_002457.1	NP_775106.2	Q52LA3.1
Branchiostoma belcheri	XM_019789564.1	XP_019641321.1	XP_019625708.1
Ciona intestinalis	XP_002119476.1	XP_004225686.3	XP_002124889.1
Saccoglossus kowalevskii	XP_006814133.1	XP_006817991.1	XP_002734202.1
Strongylocentrotus purpuratus	AAC47807.1	XP_011682985.1	XP_011662533.1
Drosophila melanogaster	NP_511170.1	NP_569958.2	NP_001284908.1
Caenorhabditis elegans	not found	NP_001023015.1	NP_001255033.1
Priapulus caudatus	XM_014810889	XP_014666074.1	XP_014662975.1
Mizuhopecten yessoensis	XP_021368124.1	XP_021347997.1	XP_021378740.1
Lingula anatina	XM_013551519	XP_013416607.1	XP_013388901.1
Stylophora pistillata	XP_022808072.1	XP_022790877.1	XP_022785302.1
Trichoplax adherens	RDD37379.1	ASM15027v1:scaffold_1:<br>7596131:7601845:1	RDD43144.1
Fusarium sp.AF-4	RSL97542.1	not found	not found
Dictyostelium discoideum	XP_628877.1	XP_637607.1	XP_643920.1
Theobroma cacao	EOY07526.1	XP_007035525.2	not found
Stentor coeruleus	OMJ80050.1	OMJ86060.1	not found

Biology Open • Supplementary information

Sequences of DNA Cassettes Used to Encode Recombinant Proteins

The following sequences were synthesized as double-stranded gBLOCK cassettes (IDT, Coralville, IA) and were then digested with the indicated restriction enzymes (at the underlined recognition sites), gel purified, and cloned into the bacterial expression plasmids described in the main manuscript in order to produce recombinant proteins for isothermal titration calorimetry.

Dm Mip130/LIN9 Myb-binding domain (digested with NdeI and XhoI)

```
CCCGGGATCCATATGACGAGAAATCGCGGCTACTCCACCTCGCTGTTGGA
GCACCTGGTGCGCCTGGAGAAGTACATTGCAGTTAAGGCGGATCGAATCC
AGCGGCTCAACAAGATGAACGGCACCGCCGAGCTGGCGATGGGCGATATG
ATAAGCCATGACGAGAATGGGGATCGCCATCGCCGACAGATTGCAGTCAA
CTTCCAGCGCCAGTATGCCTTCAACATCGTGACCATCGAGCGCATCAACG
CCGAGCTCATGTTCGAGCTCACCAAGGTGCAGGAGCTGTCCTCCAGCCTG
ACACGCAATCCCAATGTCCAGGCCATGATTTCGCCGACATATTTGCGCGA
AGAGTGCCGCGCCAAGGCGTCACAGACGGTCGACGACATCAACAAGGGCA
TGTAGCTCGAGAGATCTCCCGGG
```

Ce LIN9 Myb-binding domain (digested with NdeI and XhoI)

```
CCCGGGATCCATATGGAAATGGTTGGAAATTTCCCGCTGAAATTCCTTGT
GAATCTTGTGAAACTGACGAAATTAATTGATATCAAAAAGGGATTGATAC
GACAATTGAACGAATTGAATGCGGATGCCGAGATACAAAATATGACGTCA
GACAAATATTCGAAAGCTTTTCAGGAGAAATACGCCAAAACTATCATCGA
TCTGGAACATGTGAATCAGAATATAGATATCAATATGAATGGAATTCAAG
ATCACCACATGTATTTCTCTTCGAATGATATTTCAACGTCAAATATGAAA
CCTGAAGCAGTTAGACAAATGTGCTCTCAACAAGCTGGAAGATTTGTAGA
GCACTGTAATCAAGGATTATAGCTCGAGAGATCTCCCGGG
```

Dm LIN52 Myb-binding domain (digested with EcoRI and XhoI)

```
CCCGGGATCCGGGAATTCACGCGCAGCACCAACTACACCAGCAATCTGAC
CGATGATGATATGGTTAAGATTAATGAACTAGCCCAGCTCCCTCCCGAGG
ATCTGATCGATAAAATAAAGTCAATGCATGATGAAATTTACCAGCTGGGA
CTGCGTGAGGCAATGGAGATGACTCGTGGGAAACTGCTGGGCATCTTTGA
CCGGGATCGCGCTTAGCTCGAGAGATCTCCCGGG
```

Ce LIN52 Myb-binding domain (digested with EcoRI and XhoI)

```
CCCGGGATCCGGGAATTCTACGAATCGCCATACAAGAATATTTCGTTTCT
CAAGGAAGATGCTGTGACTGTTAATACAATGAGCCACTGCCCAGCCGACG
ATATCGCCAAGCTCATCCGAAACATTCAAAACTCGGTGTACACTCTTGGA
ATCGAAGAAGCTCGCCAGTGCCGACGTGGAAAGTTGCTCAACGTGCTGAA
ACCCACTGGCTCGTAGCTCGAGAGATCTCCCGGG
```

Biology Open • Supplementary information

WT Dm Myb MuvB-binding domain (digested with EcoRI and XhoI)

```
CCCGGGATCCGGGAATTCGTCATTGATCCCAAGTGGGCACGCGTCGCTTG
TGGCAAGTCCAGAGATCAAATGTTTATGGAGGAGCAGGCTTATGCGTGCC
TCAAAAATCTGTAGCTCGAGAGATCTCCCGGG
```

KW Dm Myb MuvB-binding domain (digested with EcoRI and XhoI)

```
CCCGGGATCCGGGAATTCGTCATTGATCCCGCGGCGGCACGCGTCGCTTG
TGGCAAGTCCAGAGATCAAATGTTTATGGAGGAGCAGGCTTATGCGTGCC
TCAAAAATCTGTAGCTCGAGAGATCTCCCGGG
```

DQ Dm Myb MuvB-binding domain (digested with EcoRI and XhoI)

```
CCCGGGATCCGGGAATTCGTCATTGATCCCAAGTGGGCACGCGTCGCTTG
TGGCAAGTCTAGAGCTGCAATGTTTATGGAGGAGCAGGCTTATGCGTGCC
TCAAAAATCTGTAGCTCGAGAGATCTCCCGGG
```

Sequences of GFP::Myb Junctions in C. elegans Transgenes

WT, KW, and DQ

```
GCT GCT GGG ATT ACA CAT GGC ATG GAC GAA CTA TAC AAA AGC CCA CAA
ala ala gly ile thr his gly met asp glu leu tyr lys ser pro gln
                GFP

AAG CTT AAG ATG GCA AGT GCG AGC ACT GAA AAC GGC GAG GAG CTG ATG
lys leu lys met ala ser ala ser thr glu gln gly glu glu leu met
                                   MYB
```

C-term

```
GCT GCT GGG ATT ACA CAT GGC ATG GAC GAA CTA TAC AAA AGC CCA CAA
ala ala gly ile thr his gly met asp glu leu tyr lys ser pro gln
                GFP

AAG CTT AAG ATG TCT GGT AGC GAT TTG AAG AGT TCG CGA ACC CAT CTC
lys leu lys Met ser gly ser asp leu lys ser ser arg thr his leu
                                       MYB
```

Biology Open • Supplementary information

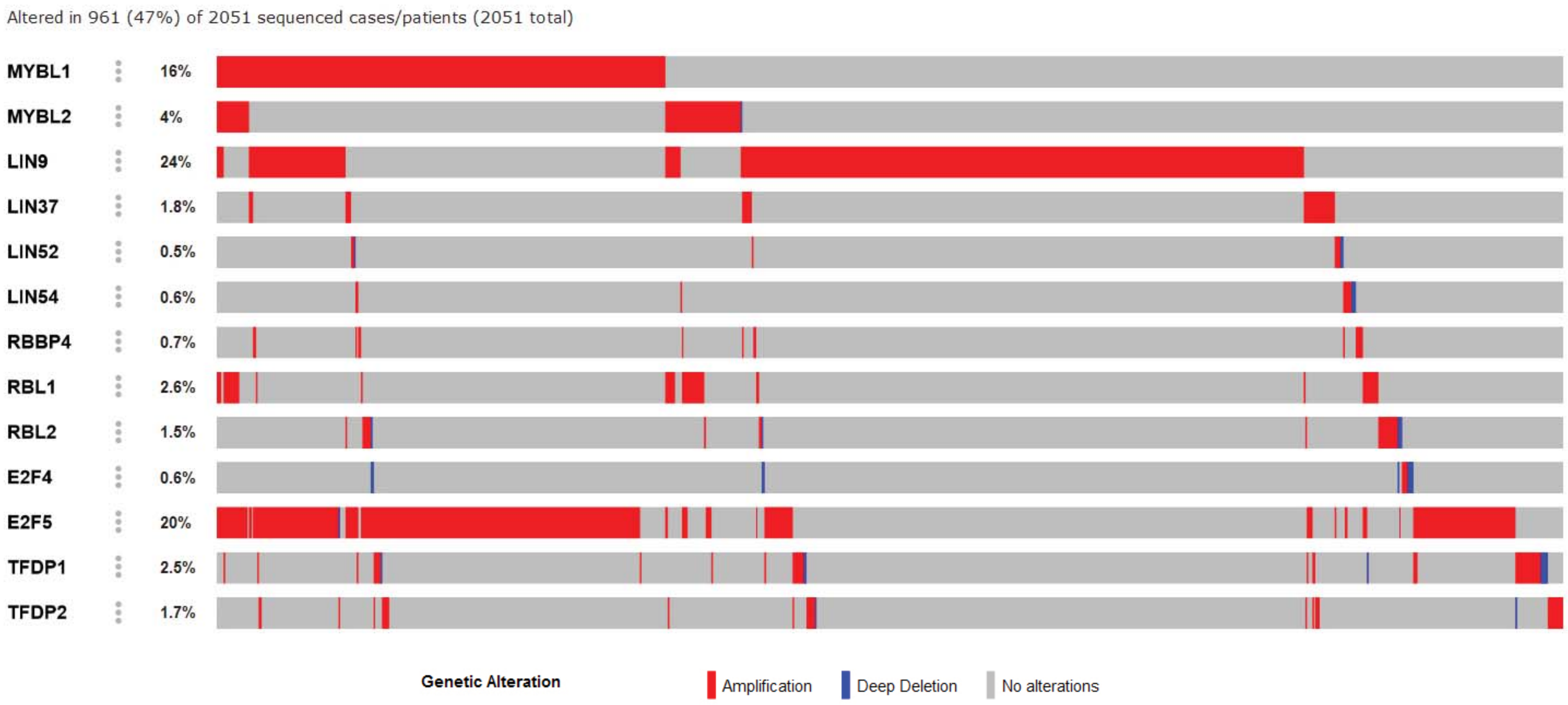

Figure S1. Genes Encoding the Myb-MuvB and DREAM Complex Proteins Are Frequently Altered in Human Breast Cancer. Publicly available cancer genome data from the METABRIC study were analyzed using the cBioPortal website [http://www.cbioportal.org/] [1,2].

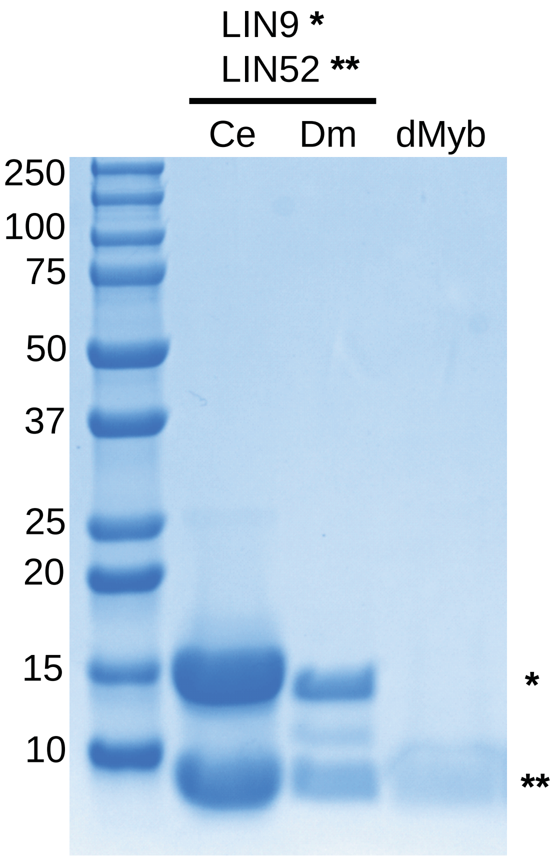

Figure S2. Proteins Used for Isothermal Calorimetry Studies. Coomassie-stained SDS-PAGE gel shows purified wild-type LIN9-LIN52 proteins (Ce: *C. elegans*, Dm: *D. melanogaster*) and purified dMyb.

Biology Open • Supplementary information

Worm Genotyping

Chromosome X: lin-15A deletion 15734037 - 15734215 (n767)

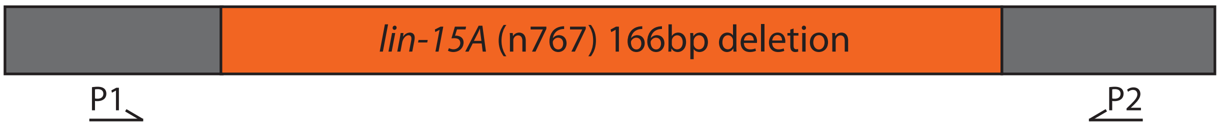

Chromosome II: region around ttTi5605 *Mos1* insertion pre-integration site

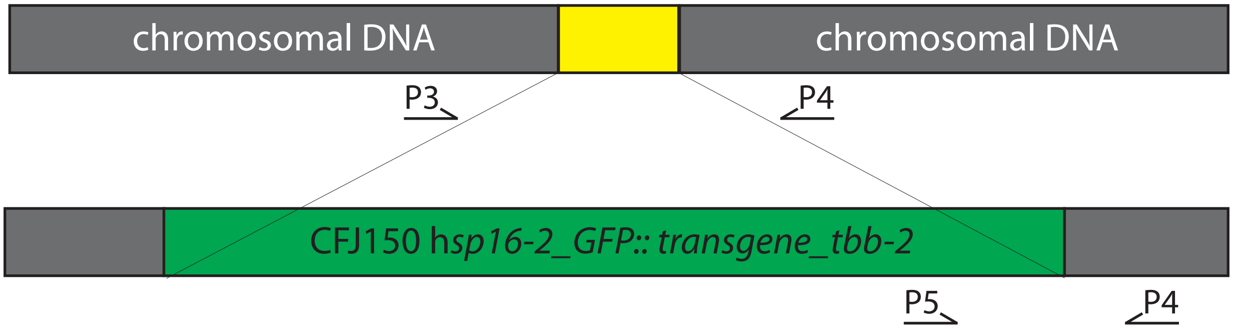

P1: gtcggcgacctgcacaaagg

P2: ccgtctccgagatgtgaaacga

P3: tgtcgaaatgtcctcctgattcca

P4: ggagttccacgcccaggaga

P5: tgcattcgaagatctgcccact

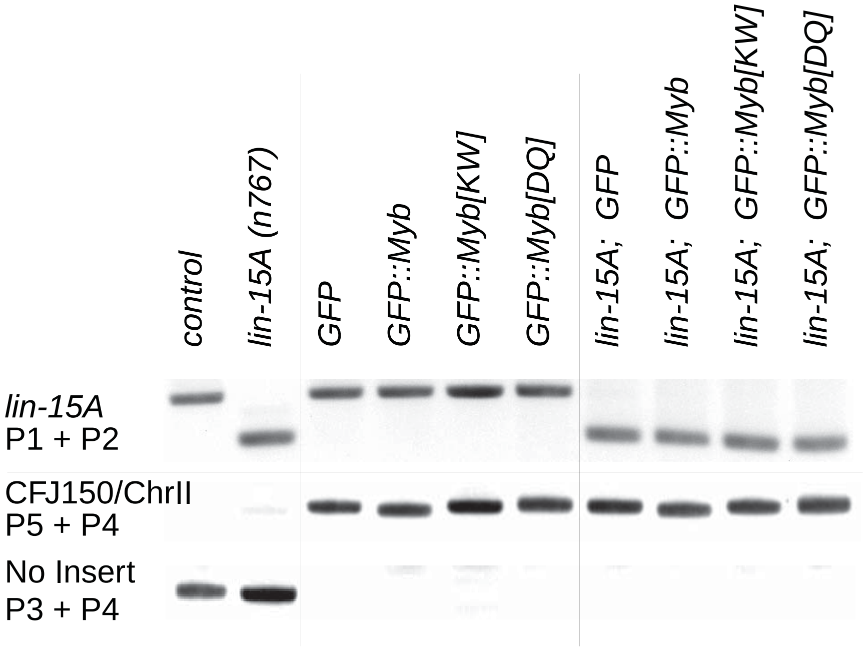

Biology Open • Supplementary information

Figure S3. PCR Genotyping of *C. elegans* Strains. Diagrams of the loci of interest, PCR primers, and predicted amplicons are shown in the top panel. The bottom panel shows a representative PCR genotyping of strains used in this paper.

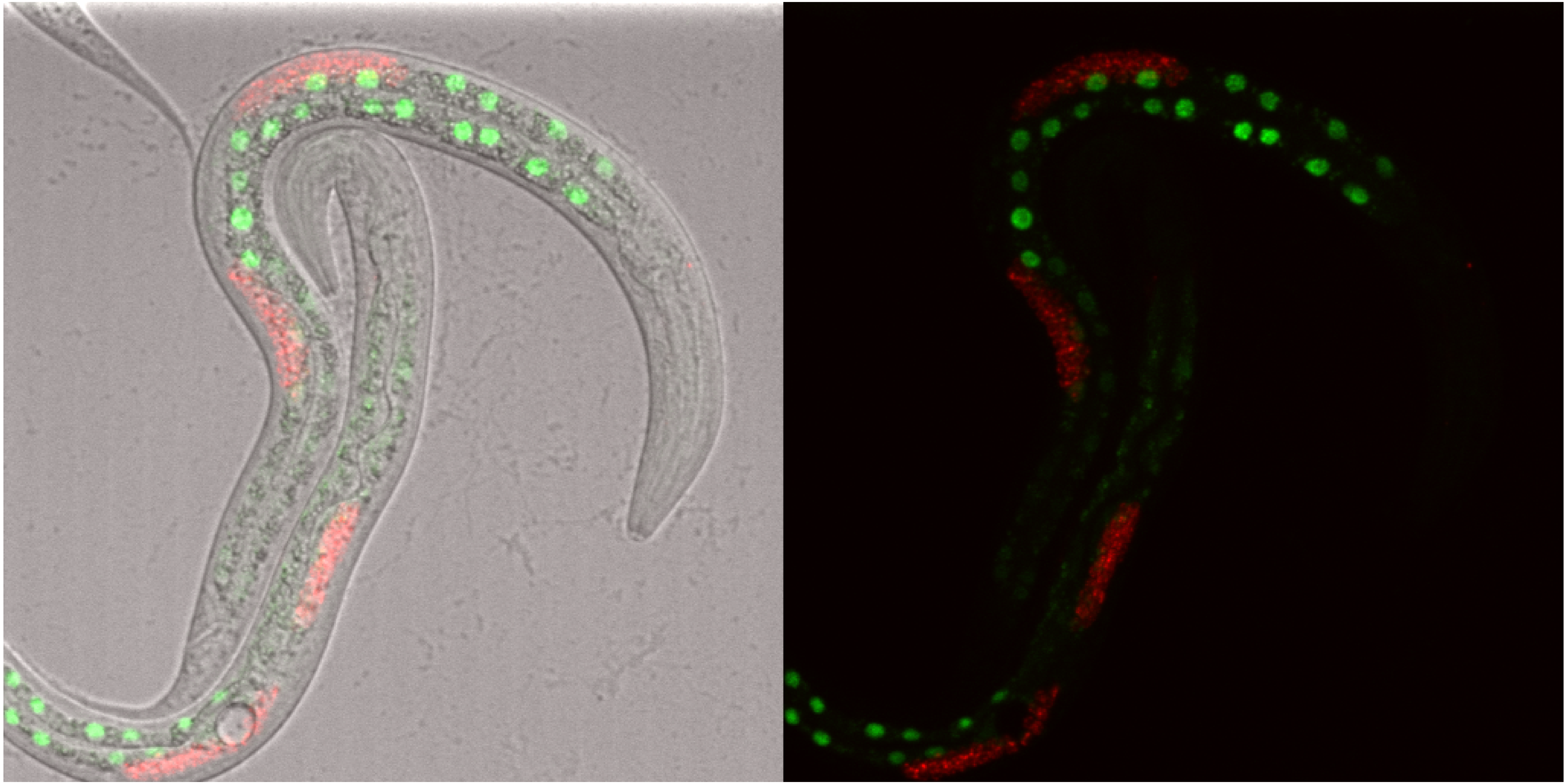

Figure S4 Part A

Biology Open • Supplementary information

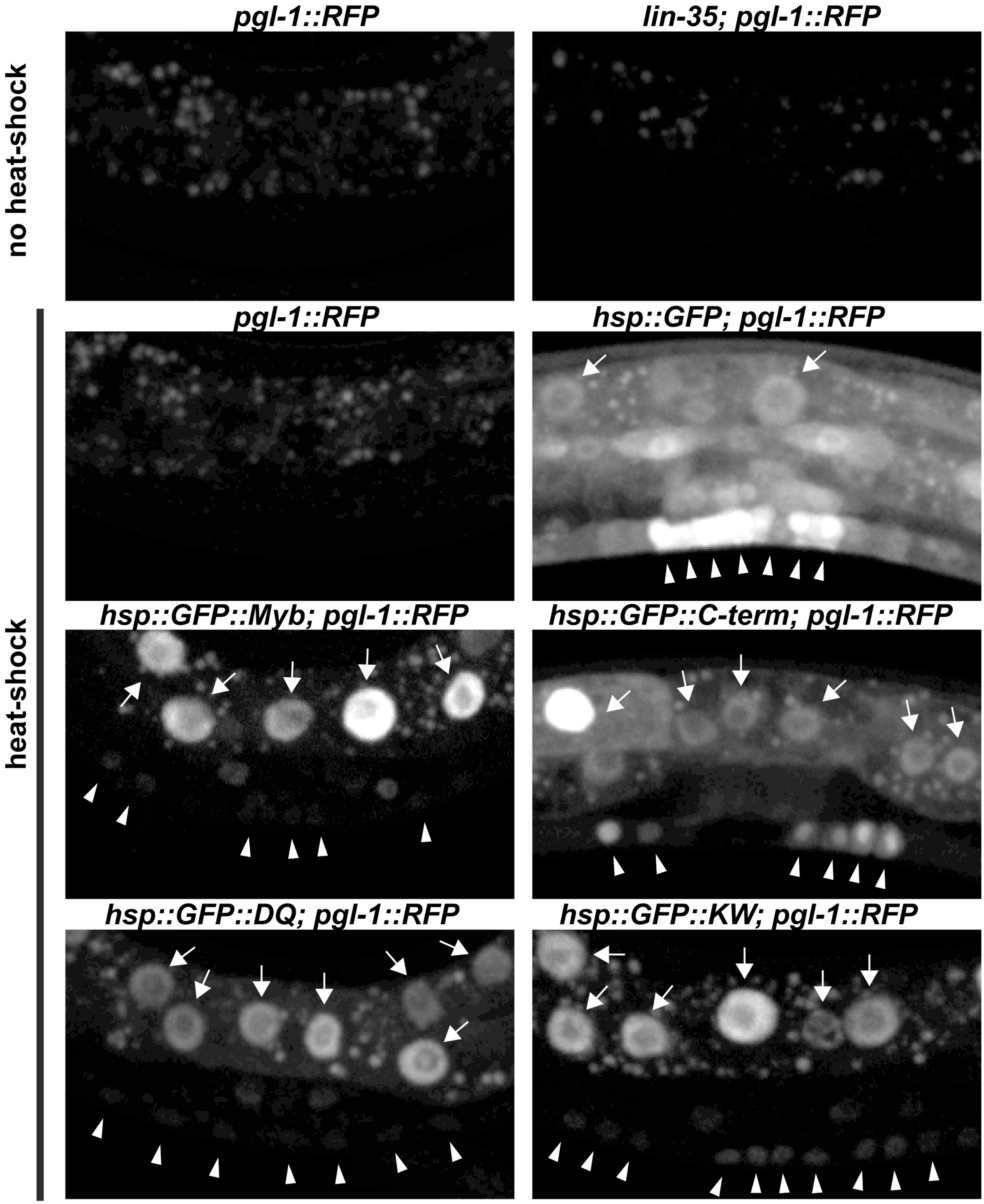

Figure S4 Part B

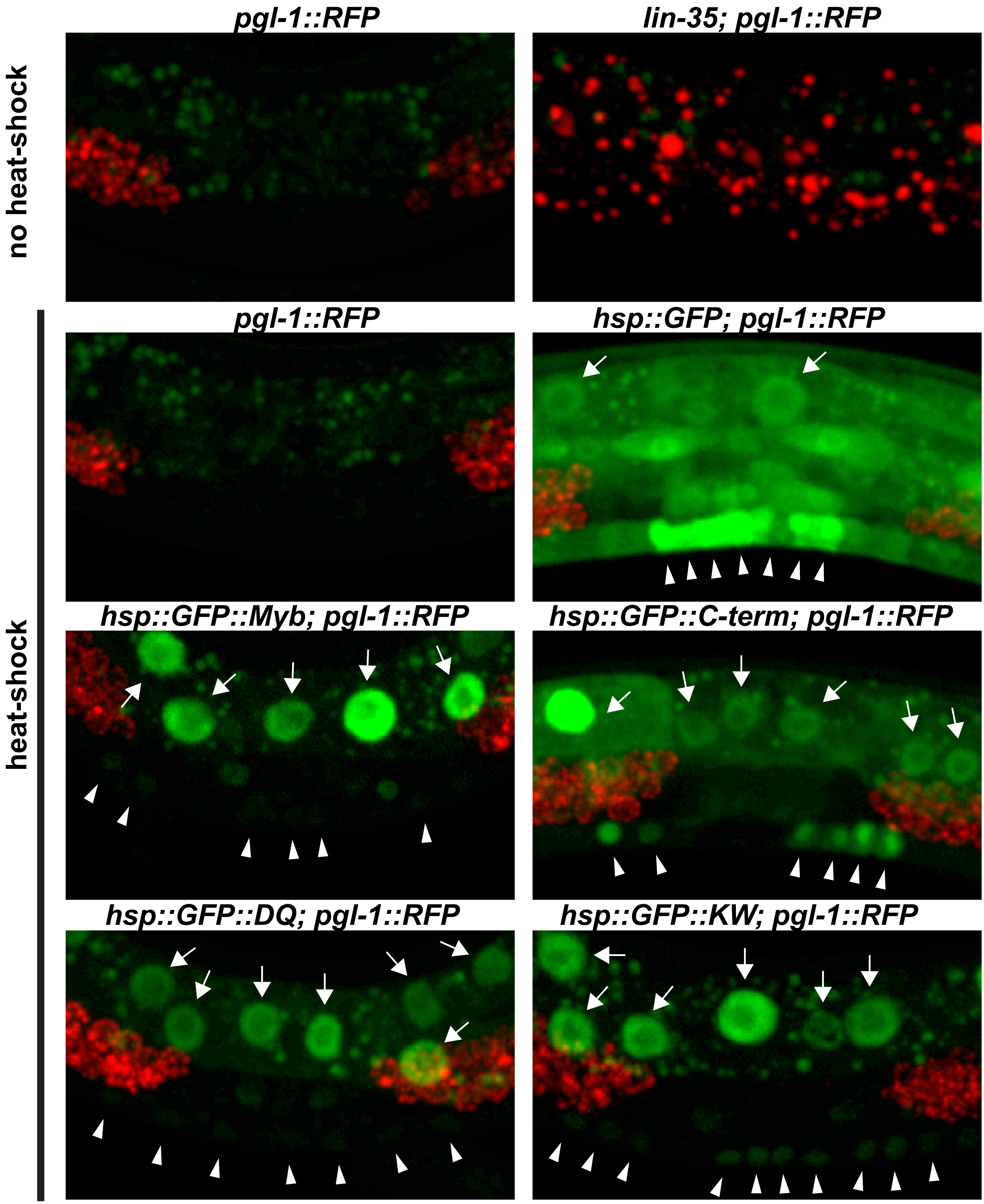

Figure S4 Part C

Figure S4. Expression of GFP and GFP::Myb Fusion Proteins in Transgenic Worms. Panel A: Photomicrographs of heat-shocked mid-L3 larvae containing an *hsp-16::GFP::Myb* transgene and a *pgl-1::RFP* reporter gene taken with a 20X objective. The left side shows GFP and RFP fluorescence superimposed on a bright-field DIC image. The right side shows only the GFP and RFP fluorescence. In each worm, GFP is visible in gut nuclei and RFP is visible in the 2 gonad arms. Panel B: Higher magnification images of heat-shocked mid-L3 larvae of the indicated genotypes showing only the GFP fluorescence. The punctate fluorescence observed in animals without heat shock is due to autofluorescent granules within gut cells. GFP-positive gut cell nuclei are indicated by arrows. GFP-positive nuclei of vulval precursor cells and adjacent hypodermal cells are indicated by arrowheads. Panel C: Same microscopic fields shown in Panel B with both GFP (green) and RFP (red) fluorescence. Note the inappropriate expression of the germline *pgl-1::RFP* reporter in gut cells in the *lin-35(n745)* mutant positive control, but not in the transgenic animals expressing GFP or GFP::Myb fusion proteins. All samples shown in Panels B and C were imaged at the same gain for a given channel.

Biology Open • Supplementary information

References

1. Gao, J. *et al.* Integrative analysis of complex cancer genomics and clinical profiles using the cBioPortal. *Sci Signal* **6**, pl1, doi:10.1126/scisignal.2004088 (2013).
2. Pereira, B. *et al.* The somatic mutation profiles of 2,433 breast cancers refines their genomic and transcriptomic landscapes. *Nat Commun* **7**, 11479, doi:10.1038/ncomms11479 (2016).

Biology Open • Supplementary information
